# Transcriptomic insights into host transcriptional manipulation by ssDNA and ssRNA viruses in the marine planktonic diatom *Chaetoceros tenuissimus*

**DOI:** 10.1016/j.virusres.2025.199605

**Published:** 2025-07-15

**Authors:** Yuki Hongo, Yuji Tomaru

**Affiliations:** a Fisheries Resources Institute, Japan Fisheries Research and Education Agency, 2-12-4 Fukuura, Kanazawa, Yokohama, Kanagawa 236-8648, Japan; b Fisheries Technology Institute, Japan Fisheries Research and Education Agency, 2-17-5 Maruishi, Hatsukaichi, Hiroshima 739-0452, Japan

**Keywords:** *Chaetoceros tenuissimus*, Diatom, Virus infection, CRESS DNA virus, *Picornavirales*, RNA sequence, gene expression

## Abstract

The marine diatom *Chaetoceros tenuissimus* coexists in the coastal ocean with two distinct viruses: a circular single-stranded DNA virus and a single-stranded RNA virus. Both viruses are capable of killing the host in vitro experiments; however, the molecular mechanisms by which host genes respond and are utilized by these viruses for replication and proliferation remain unclear. In this study, RNA-seq analyses were conducted to investigate these mechanisms, and the host transcripts specific to each virus were validated by RT-qPCR. In the early phase of infection, RNA-seq analysis revealed that gene transcription related to endocytosis, vacuolar acidification, and nitrogen metabolism was commonly upregulated. In contrast, genes related to transcription and translation were downregulated in cells inoculated with the DNA virus. In the later phase of infection, cells inoculated with the DNA and RNA viruses showed upregulation of genes related to DNA replication and packing, and autophagy, respectively. These virus-specific gene transcripts were confirmed to differ significantly by RT-qPCR. *Chaetoceros tenuissimus* has previously inserted a fragment of the DNA viral gene (EVLF) into its genome. Intriguingly, the transcription level of EVLF was upregulated in cells inoculated with the DNA virus as time progressed. Thus, regarding specific molecular responses, the DNA virus may hijack the host replication machinery to replicate its own genome, with EVLF playing a role in viral proliferation, whereas the RNA virus may hijack the autophagosome membrane to replicate its own genome, based on the observed replication of the related RNA virus at the membrane. This fundamental information provides insight into the diatom–virus infectious mechanism.

## Highlights

• The expression pattern of *C. tenuissimus* differs depending on the virus type.
• DNA virus induces host gene transcription related to DNA replication and packing.
• RNA virus induces host autophagy, wherein the membrane is a potential replication site.
• Integrated virus gene fragment in the host genome is upregulated when the DNA virus is infected.

## 1 Background

Millions of viral particles exist in the global ocean (Bergh et al., 1989). These viruses infect and lyse prokaryotic and eukaryotic organisms for recycling carbon and nutrients and then have an impact on geochemical cycles (Fuhrman, 1999; Suttle, 2007). Phytoplankton as a primary producer is also a species subject to the impact of viruses, whose abundance often simultaneously increases during phytoplankton blooms in nature and alters the population dynamics of phytoplankton (Bratbak et al., 1993; Tarutani et al., 2000; Tomaru et al., 2011). The coccolithophore *Emiliania huxleyi* exerts a cooling effect on the Earth’s climate (Charlson et al., 1987; Tyrrell et al., 1999; Westbroek et al., 1993); however, its population is often controlled by its infectious virus *Emiliania huxleyi* virus (EhV), which belongs to Nucleocytoviricota viruses (NCVs) (Bratbak et al., 1993; Wilson et al., 2002). This infectious mechanism of EhV has been well investigated at molecular and cell biology levels (Homola et al., 2024; Ku et al., 2020; Rosenwasser et al., 2014). In particular, EhV acquires host genes related to the sphingolipid biosynthesis pathway by horizontal gene transfer and induces the viral-encoded homologous pathway to facilitate its proliferation during infection (Monier et al., 2009; Rosenwasser et al., 2014). Thus, the virus possesses an advantageous survival strategy. On the other hand, phytoplankton have strategies to compete with their infectious viruses. For instance, *Ostreococcus tauri* and *O. mediterraneus* perform dynamic genomic rearrangements for acquiring resistance to their infectious viruses and can survive in the viral population (Blanc-Mathieu et al., 2017; Yau et al., 2016, 2020). Therefore, it is important to conduct molecular biological studies on microalga-virus interactions for understanding the population dynamics.

Although diatoms are one of the most prominent primary producers in the ocean, and their roles are crucial for understanding how marine ecosystems work (Field et al., 1998; Nelson et al., 1995), they are also known to be exposed to viral attacks in natural environments (Arsenieff et al., 2022). Studies have shown that diatom populations in laboratory cultures have destroyed within several days after virus inoculations (Arsenieff et al., 2022; Tomaru et al., 2015). Therefore, diatom viruses might be a factor responsible for the decreases in diatom populations; nonetheless, several studies have also reported the coexistence of diatoms and viruses during the host bloom period (Chiba et al., 2020; Tomaru et al., 2011, 2018). Because of this contradiction, there is a lack of a clear understanding of the impact of diatom viruses on host populations in natural seawaters (Bettarel et al., 2005; Kranzler et al., 2021, 2019; Tomaru et al., 2018). Accordingly, a model system, *Chaetoceros tenuissimus* and its infectious viruses, has been established to understand the interaction between the diatom and viruses. We have isolated two types of viruses from *C. tenuissimus*: a DNA virus (CtenDNAV) and an RNA virus (CtenRNAV). Each virus is further classified into distinct subtypes (type I and type II) based on differences in genome sequences (Kimura and Tomaru, 2015). CtenDNAV is a single-stranded DNA virus whose genome is circular and partially possesses a double-stranded region, and it belongs to the family *Bacilladnaviridae* of the super group circular replication-associated protein (Rep)-encoding single-strand (CRESS) DNA virus. Its genome contains at least three open reading frames (ORFs): a hypothetical protein (DVP1, BAP99809.1), a capsid protein (DVP2, BAP99810.1), and the longest ORF (446 amino acids), which shares similarity with Rep (DVP3, BAP99811.1; Kimura and Tomaru, 2015). CtenRNAV belongs to the family *Marnaviridae* within the order *Picornavirales*, and its genome encodes two ORFs: a putative replication protein (RVP1, BAP99818.1; Shirai et al., 2008) and structural polyproteins (RVP2, BAP99819.1; Munke et al., 2020). Once the virus successfully infects the host, each viral particle is accumulated in different areas in the cell, with CtenDNAV particles being located in the nucleus, whereas CtenRNAV particles being located in the cytoplasm (Kimura and Tomaru, 2015; Tomaru et al., 2011). A culture-based infectious experiment showed that although the latency periods and yields of both viruses were not significantly different, the population of host cells inoculated during the stationary phase began decreasing at an earlier period than when inoculated during the logarithmic phase (Kimura and Tomaru, 2015). This phenomenon was attributed to the fact that the virus sensitivities of the host cells might not be uniform even in vigorous cultures, and it has been confirmed that a faster growth rate of diatom populations results in a lower mortality rate (Tomaru et al., 2021). Although the viruses kill the host, there was a trace in the host genome, indicating that CtenDNAV has an evolutionary relationship with its host. It was discovered that a partial gene related to the Rep of CtenDNAV was inserted into the genome of *C. tenuissimus* strain NIES-3715 (Hongo et al., 2021). This fragment, known as endogenous virus-like fragment (EVLF), is possibly inserted by the retrotransposon in the same locus of several strains isolated around the coastal area in Japan and is transcriptionally active, although its role in viral infection remains unclear (Hongo et al., 2021).

Currently, the availability of genome information for both the diatom host and its viruses allows for detailed analysis of intracellular molecular interactions. However, fundamental questions remain about gene expression during infection specifically, how host genes respond and are exploited by a virus with a minimal genome to support infection and replication. In this study, we analyzed comprehensive host gene transcription from the onset of viral infection to host cell death, using two viruses sensitive to the host: CtenDNAV type-II (CtenDNAV-II) and CtenRNAV type-II (CtenRNAV-II). We also validated transcription levels of host genes specifically regulated during each viral infection, including EVLF, using RT-qPCR. This study proposes hypothesis to explain infection mechanisms through host genetic responses to distinct viral infections.

## 2 Methods

### 2.1 Culture conditions and cell sampling

The axenic clonal algal strain *C. tenuissimus* NIES-3715 (previous strain name: 2–10) (Shirai et al., 2008) was used as the model organism in this study, and both CtenDNAV-II and CtenRNAV-II were used as infectious viruses. Algal cultures were maintained in SWM-3 medium (salinity 30‰) supplemented with 2 nM Na2SeO3 (Imai et al., 1996) under a 12:12 h light:dark cycle of approximately 700−750 µmol of photons *m*^−2^ *s*^−1^ using white LED illumination at 25 °C. Cell concentrations were measured using an image-based cytometer (Tali® Image Cytometer, Thermo Fisher Scientific) (Tomaru and Kimura, 2016), and virus titers in the inoculated samples were estimated using the extinction dilution method (Tomaru et al., 2011).

### 2.2 Viral inocula

Early stationary growth phase cultures of *C. tenuissimus* were inoculated with CtenDNAV-II and CtenRNAV-II (0.1 % *v*^−1^), stored in the dark at 4 °C, and incubated for 7 d under the above-described growth conditions. Lysates were passed through a 0.2-µm polycarbonate membrane filter (Whatman® Nuclepore Track-Etched Membranes, Merck KGaA, Darmstadt, Germany) to remove cellular debris and stored at 4 °C until further experiments. Filtered lysates were used as the viral suspension for experimental inoculation. The CtenDNAV-II and CtenRNAV-II titers before the present experiments were 5.46 × 10^8^ and 9.68 × 10^8^ infectious units mL^−1^, respectively.

### 2.3 Virus inoculation test

Precultures for duplicate 2.1 L of culture medium at a density of 1.0 × 10^4^ cells mL^−1^ were started from 6 days before the virus inoculation under the abovementioned conditions. The densities of the duplicate cultures were 2.18 × 10^4^ and 2.22 × 10^4^ cells mL^−1^, and they were mixed just before the start of the experiment. Then, 300 mL of the cultures, containing 2.20 × 10^4^ cells mL^−1^, was poured into nine 500-mL flasks. The viral suspensions were inoculated into the culture. The inoculation volumes for both viral suspensions were 3 % *v*^−1^; thus, the multiplicities of infections (MOI) for CtenDNAV-II and CtenRNAV-II were 7.4 and 13.2, respectively. A culture inoculated with fresh medium (no virus) served as a control. Each experiment was conducted in three replicates.

The cells in 20 mL of the sample were retained onto 0.4-μm polycarbonate membrane filters (GE Healthcare) from the cultures before virus infection and after the infection at 0, 1, 3, 6, 9, 12, and 24 h, as well as the control. To prevent RNA degradation, the cells retained on the filter were immediately immersed in RNAlater (Thermo Fisher Scientific) for 5 min. After this incubation, the RNAlater was filtered out. Cells retained on the filter were frozen in liquid nitrogen and stored at −80 °C until analysis.

### 2.4 RNA extraction and sequencing

Total RNA was extracted using a TRIzol Plus RNA Purification Kit (Thermo Fisher Scientific), with PureLink DNase (Thermo Fisher Scientific) digesting any contaminating DNA, according to the manufacturer’s instructions. The quantity of total RNA was measured using a Qubit RNA HS assay kit (Thermo Fisher Scientific). cDNA libraries were constructed from 1 µg of total RNA using the TruSeq stranded mRNA prep kit (Illumina), according to the manufacturer’s instructions, and then sequenced into 150-bp paired-end reads using the Illumina HiSeqX platform.

### 2.5 Analysis of differentially expressed genes (DEGs)

Sequence reads with any adapter sequences, low-quality ends (<QV30), and unpaired reads were removed from the sequences using Trimmomatic (Bolger et al., 2014). The remaining paired-end reads generated from the transcripts of each condition were mapped to the genome sequences of *C. tenuissimus* NIES-3715 (accession no. GCA_021927905.1), CtenDNAV-II (accession no. AB971658.1), and CtenRNAV-II (accession no. AB971661.1) using HISAT2 (Kim et al., 2019). The mapped reads within the coded genes were counted as transcription levels using samtools (Li et al., 2009) and htseq-count (Putri et al., 2022). The transcription levels were normalized among the libraries using the trimmed mean of the M-value method (Robinson and Oshlack, 2010) and statistically compared between the control and virus-infected groups at each sampling time point using the edgeR package (Robinson et al., 2010) in R software ver. 3.6.0. Significant differences (*P*- values) in transcription levels were adjusted by the false discovery rate (FDR) method (Benjamini and Hochberg, 1995), where an FDR <5 % was selected as the threshold for DEGs. The normalized fragments per kilobase of exon per million mapped reads (FPKM) were calculated to represent the transcription levels of host genes using edgeR, and counts per million mapped reads (CPM) were calculated for viral genes using R.

### 2.6 Overrepresentation analysis of gene ontology and metabolic pathway

The DEGs at each sampling point were characterized by the overrepresentation analysis of gene ontology (GO) and in each Kyoto Encyclopedia of Genes and Genomes (KEGG) metabolic pathway. The overrepresentation analysis was conducted using the PANTHER classification system on the website (http://pantherdb.org/index.jsp; GO ontology database, DOI:10.5281/zenodo.10536401, released 2024–01–17). For this analysis, we used a dataset of *Thalassiosira pseudonana* proteins homologous to those of *C. tenuissimus*, as *T. pseudonana* is a closely related species with extensively annotated genomic data. To retrieve the UniProtKB ID of *T. pseudonana*, the protein of *C. tenuissimus* was assigned to that of *T. pseudonana* based on the homologous protein used in the BLASTp search. The GO overrepresentation in the gene set of DEGs between the control and virus-infected groups was analyzed using Fisher’s exact test in the biological process of GO categories. The *P*- values of the GO overrepresentation were adjusted by FDR <5 % (Benjamini and Hochberg, 1995).

The KEGG ortholog number in the KEGG metabolic pathway was assigned to the proteins of *C. tenuissimus* using BlastKOALA, which are automatic annotation servers in the KEGG website (Kanehisa et al., 2016).

### 2.7 Validation of RNA-seq transcription patterns for target genes and EVLF by RT-qPCR

Complementary DNAs were synthesized from 500 ng of the total RNA, which was extracted for constructing RNA-seq libraries, using oligo(dT)_15_ primer and SuperScript II (Thermo Fisher Scientific), according to the manufacturer’s instructions.

PCR amplification of target genes was performed in a 20 µL reaction mixture containing 1 µL of cDNA, 1 × PowerUP™ SYBR™ Green Master Mix (Thermo Fisher Scientific), and 0.2 µM of each primer. Quantitative PCR (qPCR) was conducted using a QuantStudio 3 system (Thermo Fisher Scientific) with the following cycling conditions: an initial denaturation step at 50 °C for 2 min and 95 °C for 2 min, followed by 45 cycles of 95 °C for 5 s, and 60 °C for 30 s. All reactions, including a no-template control, were performed in triplicate. A negative control using RNA as the template was also included under the same conditions. Primer sequences for target genes are listed in the Supplementary Table. The amplification of *evlf* was performed using the ctEVLF-q-v1 primers and probe, following the protocol described in our previous study (Hongo et al., 2021). The relative transcription levels of target genes were calculated using the ΔΔCt (ddCt) method, normalized to *NADH dehydrogenase* transcription, and compared to levels in cultures prior to viral infection.

## 3 Results

### 3.1 Transition of cell and virus proliferations

**Fig. 1:**
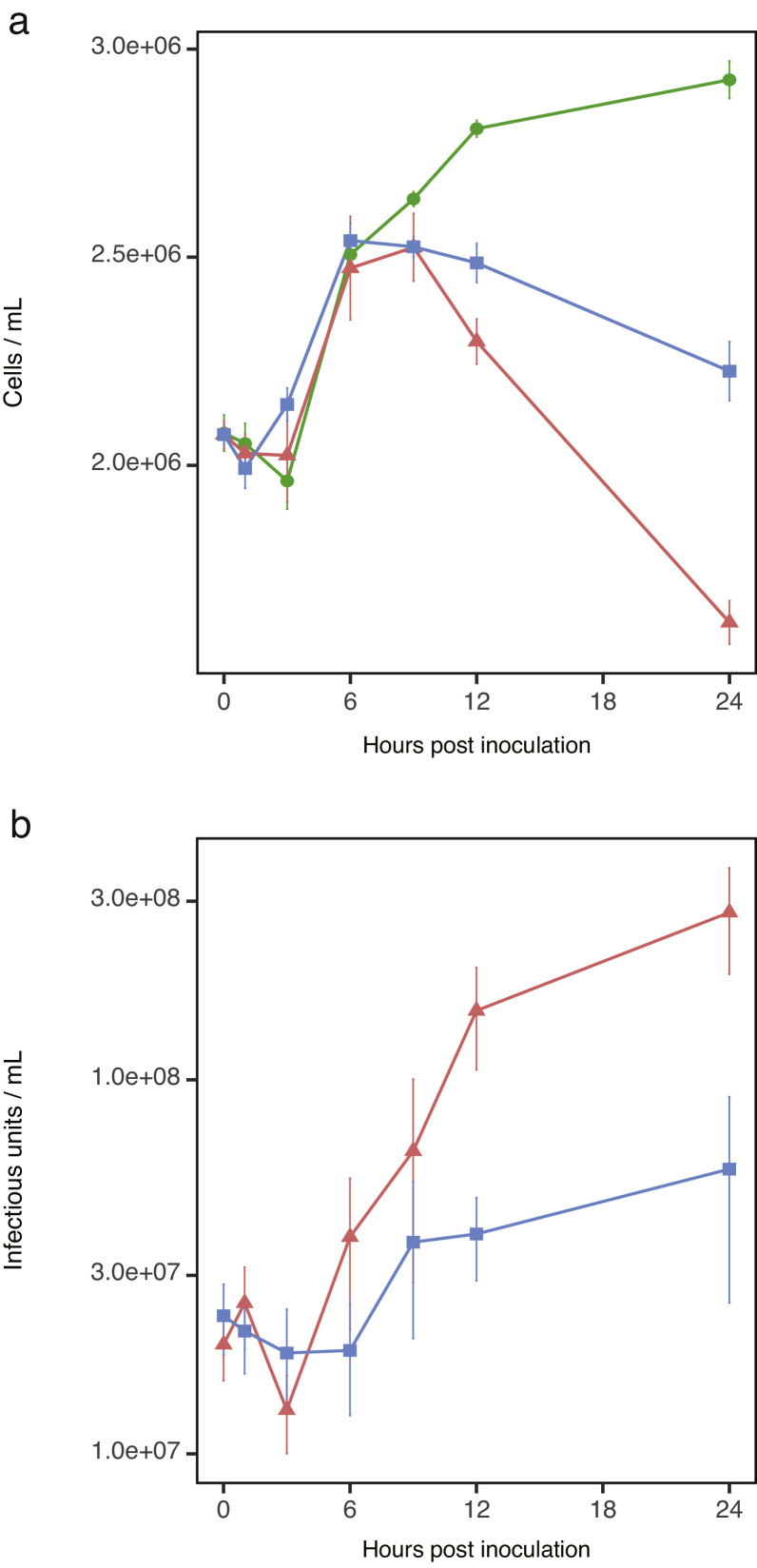
Cell concentrations (a) and viral titers (b) during the culture experiment. Green, red, and blue lines indicate control cells, CtenDNAV-II-inoculated cells, and CtenRNAV-II-inoculated cells, respectively.

CtenDNAV-II and CtenRNAV-II were inoculated into each of three culture flasks, including 2 × 10^6^ cells ml^−1^ in the growth phase. Until 3 h post-inoculation (hpi), only a few changes were observed in the cell densities compared with those in the control group (Fig. 1a). The cell densities in all culture flasks increased after 3 hpi; subsequently, those in the virus-inoculated flasks began to decline after 9 hpi, whereas those in the control flasks continued to increase (*t-test* < 0.05, Fig. 1a). After 24 hpi, the final cell densities of the control, CtenDNAV-II-, and CtenRNAV-II-inoculated flasks were 2.9 × 10^6^, 1.6 × 10^6^, and 2.2 × 10^6^ cells mL^−1^, respectively.

The viral titers in the flasks inoculated with CtenDNAV-II increased after 6 hpi compared with that at 0 hpi (*t-test* < 0.01, Fig. 1b); however, those in the flasks inoculated with CtenRNAV-II increased after 12 hpi compared with that at 0 hpi (*t-test* < 0.01, Fig. 1b).

### 3.2 Overall host–virus transcription profile with time after the inoculation of DNA and RNA viruses

Complementary DNA libraries were constructed in triplicate from the control group and the groups with inoculation of both viruses after 0, 1, 3, 6, 9, 12, and 24 h. Sequencing of the cDNA libraries using HiSeqX yielded 9–23 million paired-end reads (150 bp) per cDNA library, which were deposited in the DNA Data Bank of Japan (DDBJ) under BioProject accession number PRJDB19534.

**Fig. 2:**
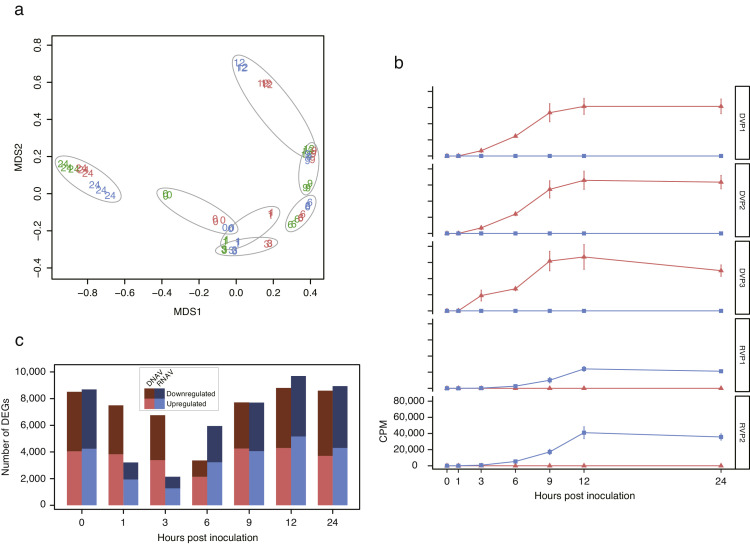
Multidimensional scaling plot of gene transcription in each culture (a). Green, red, and blue colors indicate control cells, CtenDNAV-II-inoculated cells, and CtenRNAV-II-inoculated cells, respectively. The numbers indicate hours post-inoculation (hpi). Transcription levels of the viral genes (b). Red and blue lines indicate transcription levels of CtenDNAV-II and CtenRNAV-II, respectively. Vertical and horizontal axes indicate count per million mapped reads and hpi, respectively. Number of upregulated and downregulated genes (c) at each hpi in CtenDNAV-II- and CtenRNAV-II-inoculated cells.

Using the number of reads mapped to the genomes as a measure of transcription levels, the similarity of transcription patterns across samples was visualized using multidimensional scaling (MDS, Fig. 2a). Transcription patterns were largely consistent among replicates under identical conditions; however, divergence was observed in post-inoculation samples, indicating distinct transcriptional responses to each viral inoculation (Fig. 2a). At 0 and 1 hpi, the CPM for two CtenDNAV-II genes (DVP1 and DVP2) were 0, while DVP3 showed CPM values of 0 and 118, respectively (Fig. 2b). For CtenRNAV-II, the CPM values of genes RVP1 and RVP2 were 9, 13, and 263, and 30, 39, and 741 from 0 to 3 hpi, respectively (Fig. 2b). Although transcripts of CtenDNAV-II and CtenRNAV-II began to accumulate at 3 and 6 hpi, respectively, no transcripts of CtenRNAV-II were detected in cells inoculated with CtenDNAV-II, and vice versa, indicating that cross-contamination did not occur under the experimental conditions (Fig. 2b). Host transcriptional regulation was initiated at 0 hpi following infection with both CtenDNAV-II and CtenRNAV-II (Fig. 2c). By 24 hpi, 2145–4294 and 1277–5156 genes were significantly upregulated, while 1222–4877 and 870–4645 genes were significantly downregulated in the CtenDNAV-II- and CtenRNAV-II-inoculated groups, respectively (Fig. 2c). To validate the FPKM-based transcription profiles, the relative transcription levels of 17 representative host genes and *evlf* were measured by RT-qPCR. The patterns obtained from FPKM and RT-qPCR were correlated, with coefficients ranging from 0.42 to 1.00 (Supplementary Figure). Fold changes and FPKM values for the genes focused on in this study are listed in the Supplementary Table.

### 3.3 Early response to both DNA and RNA viral infections

**Fig. 3:**
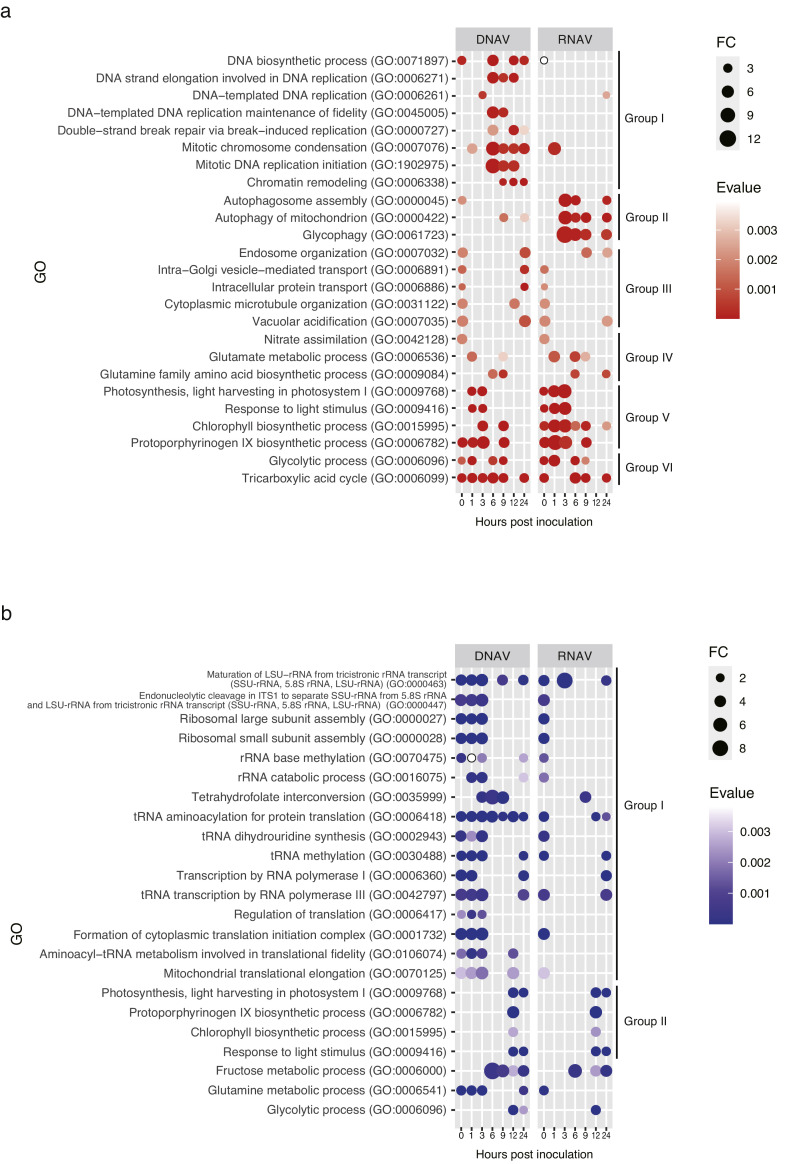
Overrepresentation of gene ontology in upregulated (a) and downregulated (b) genes during infection. The circle size and color indicate the fold change and e-value, respectively.

To characterize the regulated genes after the virus inoculations, the GO overrepresentations were detected, as depicted in Fig. 3. Immediately after the virus inoculation until 3 hpi, the GO terms related to vesicle (Group III in Fig. 3a), nitrogen metabolism (Group IV), photosynthesis (Group V), and energy production (Group VI) were enriched among the upregulated genes in cells inoculated with both viruses, whereas the GO terms related to transcription and translation were consistently overrepresented among the downregulated genes until 3 hpi in CtenDNAV-II-inoculated cells (Group I in Fig. 3b).

**Fig. 4:**
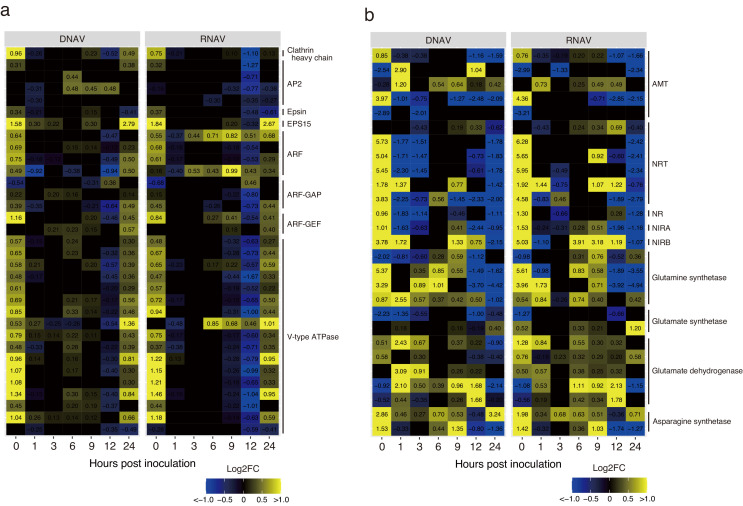
Heat map of transcriptional changes related to endocytosis and V-type ATPase (a), and nitrogen metabolism (b) in infection with each virus. The color gradient indicates the fold change, which is log_2_ transformed.

In terms of vesicle, gene transcriptions related to clathrin-mediated endocytosis were upregulated at 0 hpi (Fig. 4a), which included clathrin heavy chain (log2 fold change in CtenDNAV-II [logFCdv] = 0.96, log2 fold change in CtenRNAV-II [logFCrv] = 0.75), heterotetrameric adaptor protein (AP2, logFCdv = 0.31, logFCrv = 0.32), epsin (logFCdv = 0.34, logFCrv = 0.37), epidermal growth factor receptor substrate 15 (EPS15, logFCdv = 1.58, logFCrv = 1.84), ADP-ribosylation factors (ARFs, logFCdv = 0.49–0.75, logFCrv = 0.16–0.68), ADP-ribosylation factor GTPase-activating proteins (ARF-GAPs, logFCdv = 0.22–0.39, logFCrv = 0.15–0.45), and ADP-ribosylation factor guanine nucleotide-exchange factors (ARF-GEFs, logFCdv = 1.16, logFCrv = 0.84). Two ARF transcripts in CtenRNAV-II-inoculated cells were upregulated from 3 to 24 hpi, whereas those in CtenDNAV-II-inoculated cells were downregulated or remained significantly unchanged (Fig. 4a). Genes encoding the subunits of vacuolar-type H^+^-transporting ATPases (V-type ATPases) showed significantly higher transcription levels in the groups inoculated with both viruses at 0 hpi than in the control group (Fig. 4a, logFCdv = 0.45–1.34, logFCrv = 0.37–1.46).

For nitrogen transporters, the genes of five ammonium (AMT) and six nitrate (NRT) transporters were encoded in the genome of *C. tenuissimus* NIES-3715. The transcripts of two AMTs and five NRTs were upregulated at 0 hpi (Fig. 4b, logFCdv = 0.85–5.73, logFCrv = 0.76–6.28), and that of one NRT was continuously upregulated at 1 hpi (Fig. 4b, logFCdv = 1.37, logFCrv = 1.44). In CtenDNAV-II-inoculated cells, two AMT transcripts were upregulated at 1 hpi (Fig. 4b, logFCdv = 2.90, 1.20). Gene transcriptions encoding nitrate reduction enzymes were also upregulated at 0 hpi (Fig. 4b), including nitrate reductase (NR, logFCdv = 0.96, logFCrv = 1.30), and nitrite reductase (NIRA, logFCdv = 1.01, logFCrv = 1.53; NIRB, logFCdv = 3.78, logFCrv = 5.03). Glutamine is synthesized from glutamate by glutamine synthetase (GS), whose transcripts were significantly higher in the groups inoculated with both viruses than in the control groups at 0 hpi (Fig. 4b, GS, logFCdv = 0.87–5.37, logFCrv = 0.54–5.61). The transcript of glutamate synthetase, which converts glutamine into glutamate, was downregulated or remained significantly unchanged in CtenDNAV-II-inoculated cells (Fig. 4b), while one glutamate synthetase in CtenRNAV-II-inoculated cells was upregulated at 0 hpi (Fig. 4b, logFCrv = 0.52). Glutamate is synthesized from 2-oxoglutarate and ammonia by glutamate dehydrogenase (GDH), whose transcripts were mostly upregulated at 1 hpi in CtenDNAV-II-inoculated cells (Fig. 4b, logFCdv = 3.09). Ammonia is also used to synthesize aspartate into asparagine by asparagine synthetase (AS; Nakatsu et al., 1998), whose transcripts were upregulated at 0 hpi in cells with inoculated of both viruses (Fig. 4b, logFCdv = 1.53–2.86, logFCrv = 1.42–1.98).

### 3.4 Late response to DNA viral infection

After 3 hpi, the represented GO terms of transcripts were specifically detected in groups inoculated with each virus (Fig. 3). In CtenDNAV-II-inoculated cells, the GO terms related to DNA biosynthesis, replication, and chromatin remodeling were represented in the upregulated genes (Fig. 3a).

**Fig. 5:**
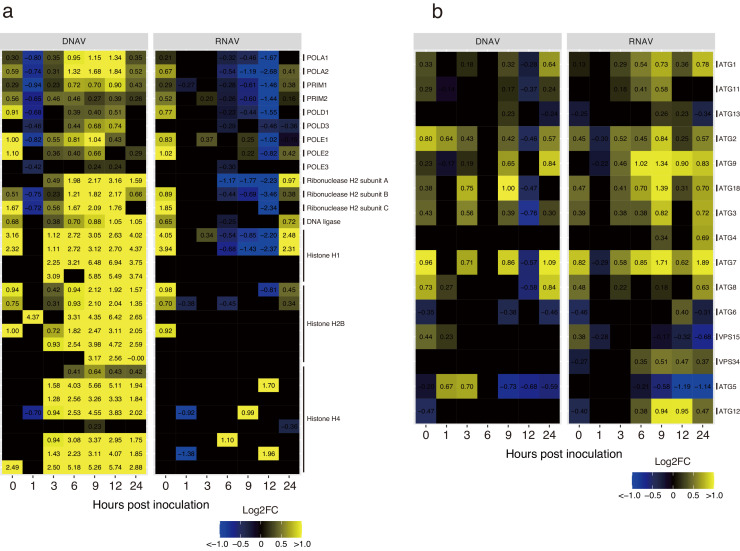
Heat map of transcriptional changes related to replication and chromatin remodeling (a), and autophagy (b) in infection with each virus. The color gradient indicates the fold change, which is log_2_ transformed.

For DNA replication, DNA polymerases in CtenDNAV-II-inoculated cells showed higher transcription levels than those in CtenRNAV-II-inoculated cells (Fig. 5a). The DNA polymerase alpha complex consists of four subunits, viz., POLA1, POLA2, DNA primase small subunit (PRIM1), and large subunit (PRIM2), which are important for the first replication step (Doublié and Zahn, 2014). These transcripts in CtenDNAV-II-inoculated cells were significantly higher than those in the control group between 3 and 24 hpi (logFCdv = 0.23–1.84, Fig. 5a), and conversely, those in CtenRNAV-II-inoculated cells were downregulated between 6 and 12 hpi (logFCrv = 0.26–2.68, Fig. 5a). During those hpi, transcripts of two subunits of DNA polymerase delta (POLD), which synthesized lagging DNA strands, and three subunits of DNA polymerase epsilon (POLE), in CtenDNAV-II-inoculated cells were upregulated (logFCdv = 0.24–1.04, Fig. 5a). Three subunits of ribonuclease H2, which remove the Okazaki fragment (Wilkins et al., 2025), and DNA ligase in CtenDNAV-II-inoculated groups were transcribed higher than those in the control group (logFCdv = 0.23–3.16 and 0.38–1.05, respectively, Fig. 5a).

In chromatin remodeling (GO:0006338) of the GO term, increased transcription levels of histones were detected in CtenDNAV-II-inoculated cells (Fig. 5a).

### 3.5 Late response to RNA viral infection

In CtenRNAV-II-inoculated cells, the GO terms related to autophagy were represented in the upregulated genes (Fig. 3a). The autophagy-related genes (ATGs) were significantly transcribed in CtenRNAV-II-inoculated cells (Fig. 5b). ATG1, 2, 7, 9, and 18 were continually upregulated from 3 to 24 hpi (Fig. 5b, logFCrv = 0.25–1.89). These genes were also upregulated in CtenDNAV-II-inoculated cells, but intermittently (Fig. 5b). Vacuolar protein sorting protein 34 (VPS34) and ATG12 were continuously upregulated from 6 to 24 hpi (Fig. 5b, logFCrv = 0.35–0.95), whereas these genes remained significantly unchanged in CtenDNAV-II-inoculated cells (Fig. 5b).

### 3.6 Transcription of EVLF in the virus-inoculated cells

**Fig. 6:**
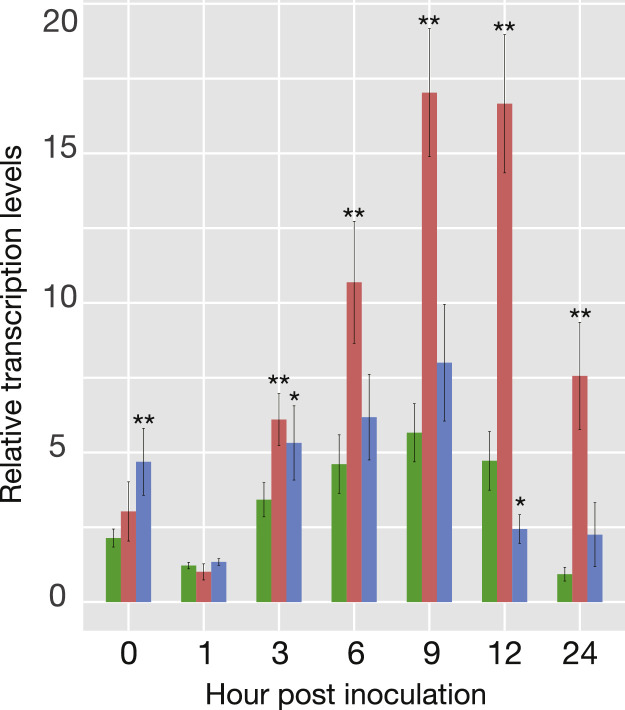
Relative transcription level of *evlf* in each culture. Green, red, and blue lines indicate control cells, CtenDNAV-II-inoculated cells, and CtenRNAV-II-inoculated cells, respectively. Asterisks denote statistically significant differences compared to the control group (*p* < 0.01 for **, *p* < 0.05 for *; *t-test*).

Although the detection of *evlf* transcripts in the RNA-seq analysis was low (Supplementary Figure), RT-qPCR detected all time points and under the experimental conditions (Fig. 6). The relative transcription levels of *evlf* in CtenDNAV-II-inoculated cells increased from 3 to 9 hpi and were significantly different from those in control cells at after 3 hpi (*t-test* < 0.01, Fig. 6), whereas those in CtenRNAV-II-inoculated cells were significantly different from those in control cells at 0 hpi (*t-test* < 0.01, Fig. 6), as well as at 3 and 12 hpi (*t-test* < 0.05, Fig. 6).

## 4 Discussion

Immediately after the inoculation of CtenDNAV-II and CtenRNAV-II into *C. tenuissimus* (0 hpi), the transcription pattern of the host differed from that in the control group, and several DEGs were also detected (Fig. 2a and c). This observation may reflect an immediate cellular response following viral recognition, as the number of viral particles per cell was sufficient to infect (MOI = 7.4 for CtenDNAV-II and 13.2 for CtenRNAV-II), and total RNA was fixed using RNA-later within 10 min of cell collection. While transcripts of CtenDNAV-II were not detected at 0 hpi in RNA-seq analysis, RT-PCR analysis detected its transcription begins 5 min after inoculation with high sensitivity (Kadono et al., 2024). Moreover, our results indicated that gene transcriptions related to endocytosis and vacuolar acidification were upregulated in cells inoculated with both viruses at this time point (Fig. 4a). Therefore, it is clear that host transcription immediately responds to the virus intrusion. AP2 and EPS15 initiate endocytic events and start the recruitment of clathrin (Kaksonen and Roux, 2018), and ARF, which belongs to the RAS superfamily of small GTPases, relates to the function of membrane vesicle transportation, whose activation is regulated by ARF-GAP and ARF-GEF (D’Souza-Schorey and Chavrier, 2006). Although the entry of virus into the cell via endocytosis was expected (Munke et al., 2022), the virus probably entered the cell through clathrin-mediated endocytosis based on the detection of the upregulation of the above-related gene transcriptions in the virus-inoculated cells. Furthermore, the transcription of V-type ATPases was upregulated, which might result in endosomal acidification after the internalization of each virus. In this case, endosomal acidification considered to function as an immune response to eliminate internalized viruses. However, RNA viruses belonging to the order *Picornavirales*, like CtenRNAV-II, utilize acidification to release their genome from the capsid (O’Donnell et al., 2008; Škubník et al., 2021). Although research on the infection mechanisms of DNA viruses belonging to the phylum *Cressdnaviricota* remains limited, endosomal acidification is also speculated to facilitate genome release during the early stage of infection in *C. tenuissimus*.

In the early stage of infection, transcriptions of the centric metabolic processes, glycolysis and the TCA cycle, were upregulated (Fig. 3a). Moreover, transcription related to chlorophyll biosynthesis and nitrogen metabolism was enhanced (Fig. 3a). Virus infection results in substantial reprogramming of host metabolism (Zimmerman et al., 2020). In particular, biosynthesis of glutamine, glutamate, and asparagine as a nitrogen fix might be improved in cells inoculated with both viruses because nitrate/nitrite transporters, ammonium transporters, glutamine synthetases, and asparagine synthase were drastically upregulated (Fig. 4b). Recent studies have demonstrated that viruses require more nitrogen (N) for their replications than phosphorus (P) (Cheng et al., 2015; Maat and Brussaard, 2016). In the case of *Phaeocystis globosa* infected with virus under N limitation, the viral burst size (number of viruses required lyse a host cell) considerably reduced by 92 % compared to 70 % under P limitation (Maat and Brussaard, 2016). The importance of N for viral replication has been demonstrated for the viral genome. For instance, a virus infected with the small green alga *O. tauri* encodes an ammonium transporter gene in its own genome, which is derived from the host through horizontal gene transfer, and improves or maintains NH_3_^+^ uptake for N requirements imposed by the viral replication (Monier et al., 2017). Although *C. tenuissimus* viruses do not encode the definitive ammonium transporter genes, they might alter N requirements in the host cell and improve the host gene expression related to nitrogen assimilation.

Upregulation of genes in the host commonly occurs during the early response to a viral infection irrespective of the type of the virus. However, GO terms related to transcription and translation were continuously represented as downregulated genes in CtenDNAV-II-inoculated cells until 3 hpi, whereas these GO terms were commonly detected in CtenRNAV-II-inoculated cells at 0 hpi (Fig. 3b). The host might suppress its own transcription to resist the viral proliferation during the early stage of infection. If this occurs, the represented GO terms in CtenRNAV-II-inoculated cells should be the same as those in CtenDNAV-II-inoculated cells, but they were not continually maintained until 3 hpi. A virus hijacks the host translation machinery and then inhibits the host protein synthesis, which is known as “shut-off,” and the virus mRNA is efficiently translated in this circumstance (Toribio and Ventoso, 2010; Walsh and Mohr, 2011). Although we have no determined evidence from the viral genes that control the host translation machinery, we speculate that CtenDNAV-II suppresses the host transcriptions related to gene translations using “shut-off” to a greater extent than CtenRNAV-II and preferentially transcribes its own genes.

In the late response, after 3 hpi, the characteristic GO terms expressing each infected virus were represented in the upregulated genes (Fig. 3a). In CtenDNAV-II-inoculated cells, the GO terms related to DNA replication and chromatin remodeling were remarkably represented after 6 hpi. These genes may contribute to the DNA replication of CtenDNAV-II rather than that of the host because the number of cells reduced, whereas that of CtenDNAV-II increased after 6 hpi (Fig. 1a and b). In general, the circular ssDNA virus genome replicates in a manner known as “rolling circle replication” (RCR) using its own Rep and host polymerases (Rosario et al., 2012). Although CtenDNAV-II may replicate its own genome in the host nucleus using RCR, the detailed underlying mechanism yet remains unknown. However, because the transcriptions of host DNA polymerases were remarkably upregulated in CtenDNAV-II-inoculated cells (Fig. 5a), it is possible that these proteins sufficiently contribute to the replication of the viral genome. In fact, geminiviruses, which belong to the CRESS-DNA viruses, infect plants and then replicate their own genome using host polymerase α (Wu et al., 2021). Similar to these genes, the transcriptions of histone proteins were also remarkably upregulated in CtenDNAV-II-inoculated cells (Fig. 5a). Geminiviruses possess Rep in their own circular ssDNA genome, and this protein binds histone H3 (Kong and Hanley-Bowdoin, 2002). The double-stranded DNA of geminiviruses forms a minichromosome in the nucleus of the infected cell, which blocks access to the overlapping viral origin of replication and the bidirectional promoters (Pilartz and Jeske, 1992). Under this situation, the interaction between Rep and histone H3 is considered to allow access to the replication machinery in the minichromosome (Kong and Hanley-Bowdoin, 2002). Therefore, when the *C. tenuissimus* DNA virus forms a minichromosome like the geminivirus, it may explain that the highly expressed histone proteins contribute to adjustment of viral replication.

CtenRNAV-II-inoculated cells represented GO terms related to autophagy in the upregulated genes. In general, autophagy functions to degrade proteins for preventing accumulation or for recycling the material in the cell, as well as to eliminate pathogens. Considering this, the genes related to autophagy should be upregulated to eliminate the infected virus in cells inoculated with both viruses as antiviral response. In fact, the upregulated transcription of these genes was also detected in CtenDNAV-II-inoculated cells; however, they were not constantly transcribed as that in CtenRNAV-II-inoculated cells (Fig. 5b). Consequently, the GO terms were highly represented in CtenRNAV-II-inoculated cells. It has been reported that enterovirus, which has a positive-sense RNA genome within the family *Picornaviridae* and is closely related to CtenRNAV-II, hijacks the secretory pathway using its polyproteins (Hsu et al., 2010) and causes the initiation of canonical autophagy (Taylor and Kirkegaard, 2007) to replicate and pack its genome in the virion attached on the autophagy-like double-membrane structures (Dahmane et al., 2022). Autophagy is strictly regulated by several ATGs. The ATG1 complex, which is composed of ATG1, 11, 13, 17, 29, and 31, is present in a specific structure, the pre-autophagosomal structure (PAS), in yeast (Suzuki et al., 2001), and the VPS34 complex, which is composed of VPS34, VPS15, ATG6, and ATG14, produces phosphatidylinositol 3-phosphate in the PAS for autophagosome formation (Mizushima et al., 2011). Moreover, the ATG8 and ATG12 conjugation systems (ATG8 conjugates with ATG3, 4, 7; ATG12 conjugates with ATG5, 7, 10, 16) function to elongate and complete the closure of the autophagosome membrane (Mizushima et al., 2011). Another complex, the ATG2 complex composed of ATG9 and 18, plays a role in tethering PAS to the endoplasmic reticulum (ER) membrane during autophagosome formation (Kotani et al., 2018). In the *C. tenuissimus* genome, partial ATG sets were detected in this study that resulted in transcription after the virus inoculation. Notably, CtenRNAV-II-inoculated cells exhibited sustained upregulation of several ATG genes (ATG1, 2, 7, 9, and 18; Fig. 5b). Although speculative, this transcriptional activation may be induced by polyproteins encoded by CtenRNAV-II, potentially to facilitate viral genome replication and encapsidation. While capsid proteins have been characterized previously (Munke et al., 2020), cleavage sites of their polyproteins and underlying mechanisms remain unclear. If the *C. tenuissimus* RNA virus replicates using the autophagosome membrane, further study is required focusing on not only the host but also the virus.

The EVLF in the genome of *C. tenuissimus* NIES-3715 showed higher transcription in CtenDNAV-II-inoculated cells than in CtenRNAV-II-inoculated cells after 3 hpi (Fig. 6). The titer of CtenDNAV-II increased after 3 hpi (Fig. 1b), thus indicating that EVLF was probably transcribed with the proliferation of CtenDNAV-II. We have speculated that EVLF functions in RNA silencing against DNA virus proliferation (Hongo et al., 2021). However, as the number of CtenDNAV-II particles increased, that of host cells decreased after 9 hpi. This implies, in contrast to our consideration, that EVLF may promote CtenDNAV-II proliferation. Because the sequence of EVLF derived from CtenDNAV varies among other strains of *C. tenuissimus* (Hongo et al., 2021), EVLF expression in other strains may differ in response to CtenDNAV-II infection, and their susceptibility to the virus may vary accordingly. Transcripts of EVLF in CtenRNAV-II-inoculated cells were also significantly higher than those in control cells at 0 and 3 hpi (Fig. 6). While it is currently difficult to draw definitive conclusions, we hypothesize that recognition of viral entry leads to increased EVLF transcription.

## 5 Conclusion

**Fig. 7:**
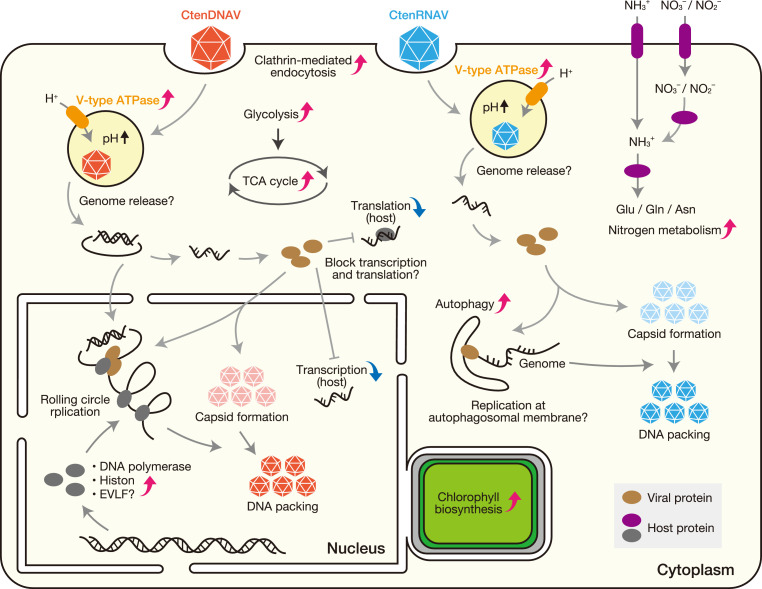
Diagrammatic overview of the hypothesized mechanism. Red upward-pointing arrowheads indicate upregulated processes, while blue downward-pointing arrowheads indicate downregulated processes.

Approximately two decades have passed since the discovery of diatom viruses, and now, transcriptomic data have allowed us to propose hypotheses regarding the infection mechanisms of each virus (Fig. 7). In the initial stage of infection, viral entry into the host cell is likely mediated by endocytosis, with subsequent endosomal acidification potentially facilitating the release of the viral genome. Moreover, both viruses reprogram host metabolism, particularly energy production and nitrogen demand. In CtenDNAV-II infection, the host’s translation machinery is controlled in the early stage, whereas genes related to replication and histone production are upregulated in the later stage, suggesting active viral proliferation. In CtenRNAV-II infection, genes related to autophagosome formation were constantly upregulated. The membrane-mediated replication mechanism, which is universally present in viruses with single-stranded RNA genomes, may also exist in diatoms. Transcription of EVLF inserted into the genome of *C. tenuissimus* was increased by CtenDNAV-II infection, causing an increase in the number of virus particles and a decrease in the number of host cells, suggesting that it may promote viral proliferation. Although these proposed mechanisms remain hypothetical and require experimental validation, we suggest that the differences in the infection process that occurs within cells for each virus must be induced by the unique genes harbored by the virus and indicate that there are suitable strategies of viral proliferation in each virus.

## CRediT authorship contribution statement

**Yuki Hongo:** Writing – review & editing, Writing – original draft, Methodology, Formal analysis, Data curation. **Yuji Tomaru:** Writing – review & editing, Writing – original draft, Supervision, Resources, Project administration, Funding acquisition, Formal analysis, Data curation.

## Declaration of competing interest

The authors declare that they have no known competing financial interests or personal relationships that could have appeared to influence the work reported in this paper.

## Data Availability

Data will be made available on request.

## Appendix Supplementary materials

Image, application 1

Image, application 2
