# Supplementary material for: Transcriptomic insights into host transcriptional manipulation by ssDNA and ssRNA viruses in the marine planktonic diatom Chaetoceros tenuissimus
Source: Virus Res. 2025 Jul 15;359:199605. doi: 10.1016/j.virusres.2025.199605 (PMC12305248; doi:10.1016/j.virusres.2025.199605)
Supplement: Supplementary file 1 [file mmc1.pdf]

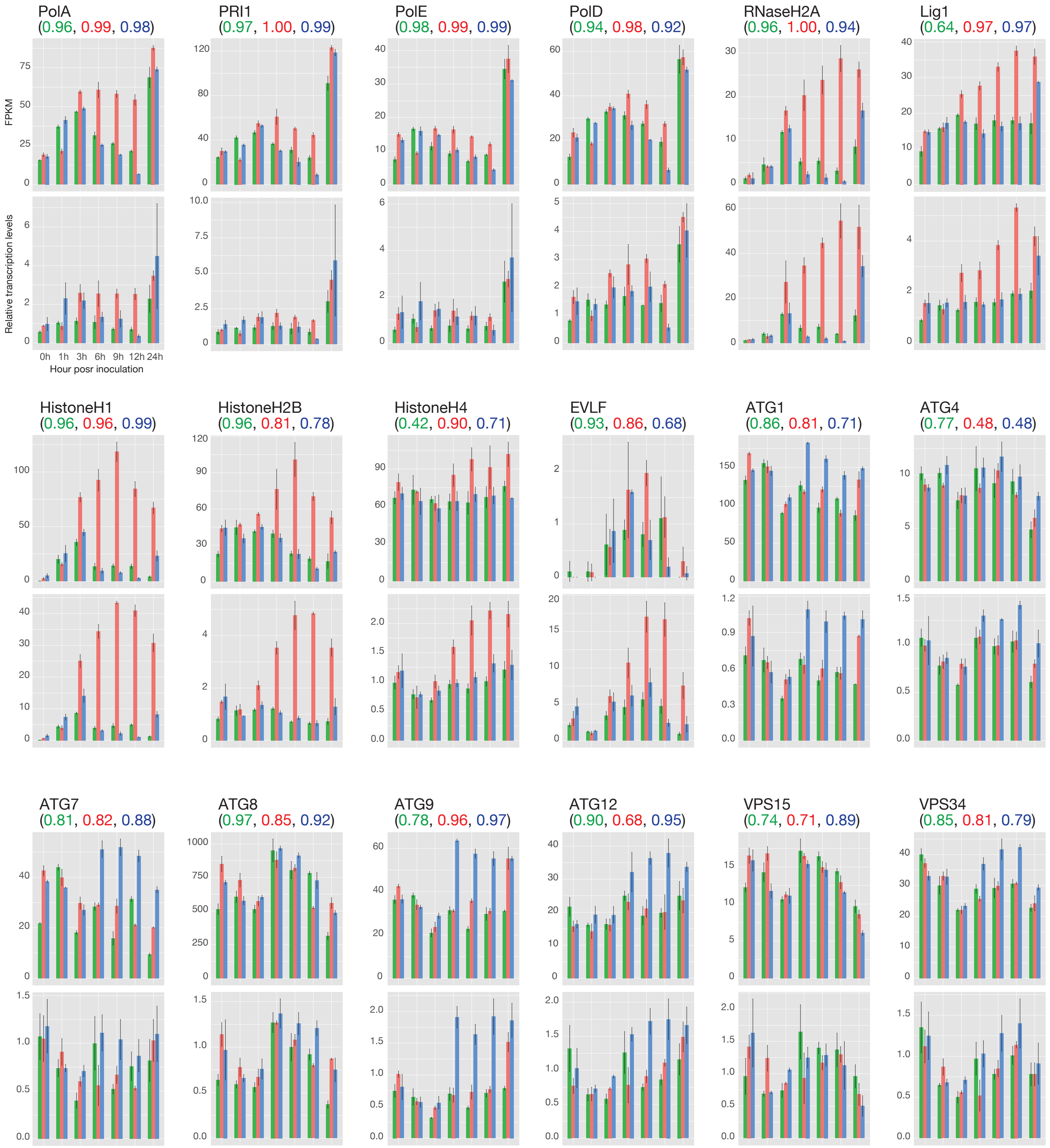

Supplementary figure: Quantitative gene transcription analysis was conducted using RNA-seq (FPKM values shown in the upper section) and RT-qPCR (relative transcription levels shown in the lower section). Eighteen genes highlighted in this study are shown. The values of the Peason correlation coefficient are indicated in parenthesis under the gene names. Colors in parentheses and bar plots represent control cells (green), CtenDNAV-II-inoculated cells (red), and CtenRNAV-II-inoculated cells (blue).
